# Daylight exposure modulates bacterial communities associated with household dust

**DOI:** 10.1186/s40168-018-0559-4

**Published:** 2018-10-18

**Authors:** Ashkaan K. Fahimipour, Erica M. Hartmann, Andrew Siemens, Jeff Kline, David A. Levin, Hannah Wilson, Clarisse M. Betancourt-Román, GZ Brown, Mark Fretz, Dale Northcutt, Kyla N. Siemens, Curtis Huttenhower, Jessica L. Green, Kevin Van Den Wymelenberg

**Affiliations:** 1 Biology and the Built Environment Center, University of Oregon, 13th Ave, Eugene, OR USA; 2 Department of Civil and Environmental Engineering, Northwestern University, Chicago, IL USA; 3 Energy Studies in Buildings Laboratory, University of Oregon, Eugene, OR USA; 4 Department of Mathematics, University of Oregon, Eugene, OR USA; 5 Department of Biostatistics, Harvard T.H. Chan School of Public Health, Boston, MA USA; 6 Santa Fe Institute, Santa Fe, NM USA

**Keywords:** Dust, Daylight, Microbiome, Built environment

## Abstract

**Background:**

Microbial communities associated with indoor dust abound in the built environment. The transmission of sunlight through windows is a key building design consideration, but the effects of light exposure on dust communities remain unclear. We report results of an experiment and computational models designed to assess the effects of light exposure and wavelengths on the structure of the dust microbiome. Specifically, we placed household dust in replicate model “rooms” with windows that transmitted visible, ultraviolet, or no light and measured taxonomic compositions, absolute abundances, and viabilities of the resulting bacterial communities.

**Results:**

Light exposure per se led to lower abundances of viable bacteria and communities that were compositionally distinct from dark rooms, suggesting preferential inactivation of some microbes over others under daylighting conditions. Differences between communities experiencing visible and ultraviolet light wavelengths were relatively minor, manifesting primarily in abundances of dead human-derived taxa. Daylighting was associated with the loss of a few numerically dominant groups of related microorganisms and apparent increases in the abundances of some rare groups, suggesting that a small number of microorganisms may have exhibited modest population growth under lighting conditions. Although biological processes like population growth on dust could have generated these patterns, we also present an alternate statistical explanation using sampling models from ecology; simulations indicate that artefactual, apparent increases in the abundances of very rare taxa may be a null expectation following the selective inactivation of dominant microorganisms in a community.

**Conclusions:**

Our experimental and simulation-based results indicate that dust contains living bacterial taxa that can be inactivated following changes in local abiotic conditions and suggest that the bactericidal potential of ordinary window-filtered sunlight may be similar to ultraviolet wavelengths across dosages that are relevant to real buildings.

**Electronic supplementary material:**

The online version of this article (10.1186/s40168-018-0559-4) contains supplementary material, which is available to authorized users.

## Background

Humans spend most of their time in the built environment [1], exposed to microbial communities associated with indoor dust. These communities are diverse [2], in part comprising putative commensal and pathogenic human-associated microorganisms [3, 4], and appear to be influenced by architectural features of the buildings they occupy [3, 5–7]. A predictive understanding of the drivers of microbial communities associated with indoor dust may therefore have relevance for human health [3, 8–13] and potential consequences for future building design and operation [7, 14].

Sunlight is a central component of architectural design [15] and has long been considered a potential buffer against the spread of pathogens in buildings [16–20] due to its potential bactericidal effects [21]. Culture-based investigations of a small number of bacterial taxa have indicated that exposure to light, and especially ultraviolet (UV) wavelengths [16, 22–25], can inactivate many microorganisms and therefore potentially reduce dust microbial community viability. It has, however, been difficult to extend these findings to dust communities in real buildings since ordinary windows transmit visible light and *block* most ultraviolet wavelengths [26]. Changes in lighting also typically co-occur with changes in human occupancy, temperature, and humidity conditions. A coherent understanding of when mortality of viable microorganisms does or does not occur in dust, and whether different light exposures influence these processes at the microbial community scale, is still lacking.

One impediment to a comprehensive understanding of indoor microbiome community structure is that controlled and manipulable built environment experiments are logistically challenging and rarely achievable. As a result, indoor microbiome research has primarily relied on non-invasive in situ observational sampling. These studies have revealed associations between abiotic features like humidity, temperature, and ventilation, and the structure of indoor microbial communities [2, 3, 5, 27–29]. However, parsing the effects of the numerous covarying abiotic and biotic factors that are hypothesized to influence indoor microbial communities remains a significant challenge for observational studies in occupied buildings [28]; manipulative experiments are still needed to disentangle the relative contributions of these factors toward shaping the built environment microbiome [6].

**Fig. 1:**
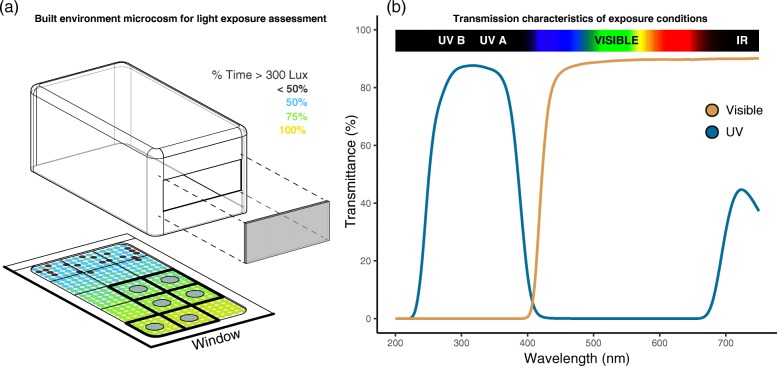
Schematic of experimental system and light treatments. **a** Diagram of a microcosm showing the floor plate, the box comprising the walls and roof, and the window opening and glazing. The floor of the box shows a simulation of the total visible light exposure during the study period in a real-world space of the same proportions. The color scale indicates the percentage of total time (during daylight hours) each point receives at least 300 lx—an illumination target level common for office-type spaces. For representation purposes only, these values were calculated using RADIANCE daylight simulation software [81]. These values are within the range of typical values found in daylit buildings. Thick outlines and circles on the grid mark the locations of the six replicate dust communities within each microcosm. **b** Transmittance (%) of different light wavelengths through the visible (gold) and ultraviolet (blue) light treatment glass pane across the range of UV and visible light wavelengths. Both glass treatments permitted the transmittance of some near-infrared (dark red bands) and infrared (black bands) light wavelengths

Microcosms—small artificial habitats—have been central in experimentally testing otherwise intractable community-level hypotheses in ecology and microbiome research [30–32], due to the ability to manage and replicate environmental conditions in these systems. Here, we combine a controlled microcosm experiment with ecological sampling models to test the hypotheses that light exposure (i) leads to compositionally distinct dust bacterial communities, (ii) reduces the total abundance of living bacteria compared to dust experiencing darkness, and (iii) impacts phylogenetically related taxa in similar ways. As a secondary goal, we sought to determine whether these daylighting impacts depended on the transmittance of ultraviolet compared to visible light wavelengths. Finally, we developed an ecological sampling model in order to evaluate observed changes in bacterial community structure against null expectations [33, 34], as a tool for generating hypotheses about the mechanisms underlying experimental outcomes. To accomplish these aims, we established an array of small climate-controlled built environment “rooms” and inoculated them with dust collected from residential homes in Eugene, OR, USA. A window was installed in each microcosm that filtered sunlight passing into the rooms and created a natural gradient of light exposures of either mostly visible or ultraviolet light. Replicate dust communities were positioned within each microcosm (Fig. 1a), and the Illumina MiSeq platform was used to sequence amplified fragments of the 16S rRNA gene which, together with real-time quantitative polymerase chain reaction (qPCR) and propidium monoazide (PMA) treatment, allowed us to measure taxonomic compositions, total abundances, and viabilities of the resulting bacterial communities after a 90-day period and to compare these emergent community features to those from dust in dark rooms.

## Methods

### Experimental design

We constructed eleven identical built environment microcosms that simulated lighting, reflectance, temperature, and humidity conditions in a typical indoor room. These boxes were 1:32 scale models of a room measuring 4.3 m wide, 7.9 m deep, and 3.3 m tall, with a single 3.5 m × 1.2 m view window and 1 m sill—dimensions and proportions that are well within those of typical residential and non-residential rooms. These microcosms were positioned in south-facing building openings with very little solar obstruction such that the window of each microcosm was exposed to the outside and were sealed to their base plates with rubber gasketing to prevent air exchange. The microcosm floors were demarcated by a 3 × 5 grid (Fig. 1a).

One of three glazing treatments was applied to the windows of nine microcosms, transmitting either mostly (i) visible, (ii) ultraviolet, or (iii) no light (i.e., dark; an aluminum plate). The visible-transmitting glass has a spectral profile intended to represent ordinary architectural glass used in buildings [26], blocking a large portion of UVA and UVB but admitting most visible and near infrared (Fig. 1b). The UV-transmitting glass has the opposite profile, admitting most UVA and UVB radiation but blocking most visible and near-infrared (Fig. 1b). This served two purposes. First, it allowed us to compare dust community structures in rooms that are daylit to those that are not (i.e., contrasts between visible light and dark dust communities). Second, it allowed us to determine the extent to which ultraviolet wavelengths were responsible for observed patterns in microbiome structure when compared to rooms receiving visible light. This is an important distinction since prior work has suggested a strong effect of ultraviolet light wavelengths on mortality of bacterial taxa [16].

Two additional microcosms were outfitted with light sensors within each cell of the 3 × 5 grid: one microcosm for visible (LI-COR 210SZ, Lincoln, Nebraska, USA) and UV wavelengths (Apogee SU-100, Logan, Utah, USA), in order to measure hourly visible and UV light dosages throughout the microcosms. One additional sensor of each type was placed on the roof of the building to monitor total exterior light. Temperature sensors (Onset, Bourne, Massachusetts, USA) were installed in the ceiling of each microcosm to monitor ambient conditions to confirm that they held within ranges observed in buildings. Microcosms were placed in plywood enclosures with thermostatically controlled climate systems and small fans for air mixing to provide additional temperature regulation. Temperatures were maintained between 18.19 and 22.34 ^∘^C for the duration of the experiment, typical of conditions in buildings, with an average of 20.28 ^∘^C. We confirmed that neither maximum nor minimum daily temperatures varied significantly between rooms, regardless of light treatment, using linear mixed effects models (*P*=0.58, *P*=0.09 respectively) [35]. Relative humidities in all microcosms were maintained between 23 and 64% for the duration of the experiment. This range is consistent with real-world spaces according to design standards for both winter and summer periods [36].

Microcosms produced an average visible light ratio of interior to available exterior light of *ca.* 2.7% over the course of the experiment. As a reference, schools and classrooms are often designed for a ratio of 2 to 4%, whereas buildings like warehouses typically range from 2 to 10% [37]. Thus, the distribution of daylight achieved in our microcosms was consistent with real-world spaces. The ultraviolet microcosms therefore experienced light conditions consistent with what would be expected if architectural glazings admitted these wavelengths.

Dust was collected from seven volunteer residential single family homes in Eugene, OR, USA. Residents were instructed to use personal vacuum cleaners to collect and pool dust from every room of their homes. The collected dust was mixed and homogenized using scissors in a dark laboratory. Six replicate dust samples weighing 0.25 g were collected from the homogenized dust pool and applied in a thin layer to individual sterile petri dishes for each microcosm. We demonstrate that repeated samples from this homogenized dust pool produce relatively similar bacterial communities in Additional file 1. Microcosms were sterilized with ethanol prior to the start of the experiment, and the petri dishes were placed on the delineating grid (Fig. 1a) in each of nine microcosms (6 dust inocula × 3 microcosms per treatment × 3 treatments = 54 bacterial communities in total). The experiment was conducted from December 21, 2015, to March 18, 2016.

### Sample collection and molecular analysis

After a 90-day exposure period, the dust samples were collected from all microcosms and subdivided into two equal aliquots of 0.125 g. A 90-day period was chosen based on estimated residence times for dust particles in real buildings with normal cleaning frequencies [38] and because it allowed us to characterize long-term changes in the dust microbiome relative to bacterial generation times. One of these dust aliquots was placed into a 15-mL tube for propidium monoazide (PMA) treatment, to separate the viable from the total (i.e., the combined living and dead) bacterial community [39]; the other did not receive PMA treatment and instead was extracted using the MoBio PowerSoil DNA Extraction Kit (MoBio, Carlsbad, CA, USA). Upon photo activation, PMA links to extracellular DNA, precluding amplification by polymerase chain reaction [39, 40]. Two milliliters of 1x phosphate-buffered saline (PBS) was added to each 15-mL tube to suspend the dust. Each tube received 5 *μ*L of 20 mM PMA (Biotium, Fremont, CA, USA) based on the manufacturer’s instructions, was vortexed for 5 s, placed in the dark for 5 min, and finally placed on a bed of ice for photo activation. PMA was activated using two 500-W halogen lamps placed above the samples for 15 min. At the 5- and 10-min marks, tubes were vortexed and placed back on the bed of ice. After PMA activation by light treatment, an additional 2-mL of PBS was added to each sample. The samples were then centrifuged (Eppendorf 5810R) at 3000 rpm for 10 min and the supernatant removed; the remaining bolus of dust was extracted from the tube and transferred to a MoBio PowerLyzer Glass Bead Tube for DNA extraction.

Both PMA- and non-PMA-treated DNA were amplified in a PCR enrichment of the V3 and V4 (319F-806R) regions of the 16S rRNA gene following the protocol described by Kembel et al. [41]: PCRs were purified with a bead-based DNA clean-up protocol using Mag-Bind RxnPure Plus (Omega Bio-tek, Norcross, GA, USA), quantified using Quant-iT dsDNA assay kit, and pooled with equal concentrations of amplicons using an Eppendorf epMotion 5075 robot. The DNA from all samples was manually extracted using the MoBio PowerLyzer PowerSoil DNA Isolation Kit according to the manufacturer’s instructions with the following modifications: 0.125 ± 0.01 g of dust sample was used, 1 mL of bead solution was used, samples were vortexed using a BioSpec Mini-BeadBeater 96 for 1 min, and solutions C4 and C5 were substituted for PW3 and PW4/PW5 solutions from the same manufacturer’s PowerWater DNA isolation kit as in [41]. Libraries were sequenced on an Illumina MiSeq generating 250 bp paired end reads.

We estimated the total counts of 16S rRNA gene copies per milligram of dust (a proxy for absolute bacterial abundances) of living and total communities using real-time quantitative PCR (qPCR; Applied Biosystems StepOnePlus System). The reaction mixture (50 *μ*L) contained ABS PowerUp SYBR Green PCR Master Mix (25 *μ*L), 10 *μ*M Total Bacteria F SYBR Primer 5 ^′^-gtgStgcaYggYtgtcgtca-3 ^′^ (2 *μ*L), 10 *μ*M Total Bacteria R SYBR Primer 5 ^′^-acgtcRtccMcaccttcctc-3 ^′^ (2 *μ*L), PCR grade water (16 *μ*L), and 5 *μ*L of 1:10 diluted DNA template [42]. The plate was prepared using an Eppendorf epMotion 5075 robot. The thermocycling program was as follows: initial denaturation for 2 min at 50 ^∘^C, 2 min at 95 ^∘^C; 40 cycles of 15 s at 95 ^∘^C, 15 s at 60 ^∘^C, and 60 sec at 72 ^∘^C; followed by a melt curve in the range of 60 ^∘^C to 95 ^∘^C. Standard curves were generated using serial-dilutions of synthetic 167 bp gBlocks Gene Fragments (Integrated DNA Technologies, Coralville, Iowa, USA) with known gene sequence copy numbers.

### Statistical analyses

Raw Illumina sequence data were filtered, trimmed, and denoised using the DADA2 v1.7.0 statistical inference algorithm [43, 44], which identifies ribosomal sequence variants (RSVs) and has the benefit of fewer spurious sequences compared to cluster-based approaches used for inferring operational taxonomic units. Forward reads were truncated at 200 nt, and each read was required to have fewer than two expected errors based on quality scores. Taxonomy was assigned to RSVs using the RDP Bayesian classifier implemented in DADA2 against the Silva [45] version 128 reference database, with a 75% bootstrapped threshold for retaining classifications. Prior to analyses, we removed variants classified as mitochondria or chloroplasts, as well as those that were unclassified beyond the kingdom level. RSV counts were normalized by rarefying the dataset to a sequencing depth of 50,000 sequences per sample and converted to absolute abundances (16S rRNA gene copies × mg ^−1^ dust) by scaling relative normalized RSV counts in each community by estimates of total bacterial abundance per milligram dust generated by qPCR assays [46]. To remove putative contaminants, we followed the suggestion of Nguyen et al. [47] and subtracted the number of sequences of each RSV present in negative PCR and DNA extraction kit controls from the sequence counts in experimental samples; this approach eliminated only four rare RSVs.

Quantitative bacterial community dissimilarities, or *β*-diversities, were calculated using the Canberra distance measure [48] and log101+*x*-transformed absolute RSV abundances. The effects of different light treatments on the community compositions of dust were quantified using a permutational multivariate analysis of variance (PERMANOVA). Pairwise contrasts between treatment groups were accomplished by performing PERMANOVA analyses with 10,000 matrix permutations for each pair of factor levels and adjusting *P* values for multiple comparisons using the Benjamini-Hochberg procedure [49]. Differences in group variances were tested using a multivariate homogeneity of groups dispersions analysis (permdisp2 procedure; [50]) with ANOVA and Tukey’s post hoc test. Differences between qPCR-based estimates of total and living bacterial abundances between communities experiencing visible, ultraviolet, or no light were assessed using ANOVA and Tukey’s post hoc test. All analyses were conducted with the statistical programming language, R [51].

Community dissimilarities were visualized using t-distributed Stochastic Neighbor Embedding (t-SNE) [52, 53]. t-SNE is a nonlinear embedding technique that is useful for visualizing high-dimensional data that lie near a low-dimensional manifold [52]; this visualization technique was selected because of a small number of variants with large absolute abundances (see Results) that yielded uninformative arch effects [54, 55] when *β*-diversities were visualized with unconstrained principal coordinates analysis (PCoA). We accomplished t-SNE visualization by initializing the Barnes-Hut implementation of the algorithm [53] in the Rtsne package using point coordinates generated by PCoA.

### Bacterial source tracking

We classified the types of living and dead microbial communities that remained in dust following the 90-day exposures using a Bayesian source tracking classifier (SourceTracker v1.0.1; [56]). Our goal was to estimate the relative contributions of human- and environmentally derived microbiomes to each dust community that persisted after light treatment. We amassed a training dataset comprising local human and environmental microbiomes that, like our dust samples, were collected in or near Eugene, OR, USA. Human microbiome training data included bacterial communities from a set of human arm and leg skin swabs (*N*=94) from local volunteers and a subset of fecal communities from the American Gut Project’s [57] Oregon residents (*N*=83). Environmental microbiome training data included outdoor air settling dishes (*N*=27) placed outside local residential homes and a set of soil cores (*N*=21) collected from an Oregon forest for the Earth Microbiome Project [58]. Details on the datasets used for source tracking are provided in Additional file 1.

To account for variation in sample collection, processing, and sequencing depth among individual studies and sequencing runs, the final collated training dataset used for source tracking was aggregated at the level of bacterial genus and rarefied to a depth of 2500 sequences per sample; taxa whose genus-level classification did not meet the 75% bootstrap threshold against the Silva version 128 reference database were aggregated at the next highest taxonomic level. The trained model was then tested on experimental samples that were aggregated using the same procedure, generating coarser-grained predictions than RSV-level analyses.

### Phylogenetic analysis

We used phylogenetic tree-based sparse linear discriminant analysis (sLDA) as a feature selection tool, to identify whether individual RSVs or groups of related RSVs discriminated between experimental dust communities under different lighting regimes. The details of this analysis are described by Fukuyama et al. [59] and summarized below. Briefly, we created a de novo phylogenetic tree of RSVs using a maximum likelihood GTR+ Gamma phylogenetic model in FastTree [60] following Callahan et al. [44]. The tree was used to generate two feature sets: one comprising log101+*x*-transformed absolute abundances of each RSV leaf, and another comprising each node in the tree. For the latter set, values associated with each node were log101+*x*-transformed summed abundances of all descending RSV leaves. These were scaled and used as input to the implementation of sLDA in the sparseLDA package; the optimal number of model predictors and sparsity parameter were determined by five repeats of fivefold cross-validation. This approach ignores branch lengths and instead incorporates phylogenetic information by employing a sparsity constraint that allows the simultaneous modeling and selection of leaf and node features with strongly covarying feature values [59].

### Ecological sampling theory

We build upon theory developed by Klein et al. [61] and develop a computational null model [33, 34] that predicts qualitative differences in RSV abundance patterns following the simulated loss of a small number of abundant “light-sensitive” bacteria. The model predicts changes in the detection rates, and therefore the *apparent* abundances, of taxa in pairs of nearly identical communities where one has lost a small number of abundant community members. These changes are said to be apparent because the underlying communities are otherwise identical; differences in RSV abundances only seem to occur as a result of the loss of highly abundant taxa, which relaxes limitations on the detection rates of all others [62]. The primary goal of this modeling procedure was to generate null expectations regarding those biases and to gain intuition into how they may influence observations of dust communities following light treatment.

Our model is derived from two community scale patterns. Analogous to the species abundance distribution in ecology [63], we first assumed a sequence abundance distribution (SAD) describing the abundances $(\chi _{i})_{i=1}^{S}$ of 16S rRNA gene copies per milligram dust originating from the living and dead cells of *S* bacterial taxa in a community. We assumed a lognormal distribution for this SAD, which is commonly used in ecological models [63], whence $(\chi)_{i = 1}^{S}$ is a random sample from Lognormal(*μ*,*σ*). Second, we assumed that the fraction of the *χ*_*i*_ gene copies which originate from living cells is given by the logistic function

1 $$\begin{array}{@{}rcl@{}} \alpha(\chi_{i}) = \frac{\lambda - \phi}{1 + e^{-k (\chi - \chi_{0})}} + \phi, \end{array} $$

where *ϕ* and *λ* are the minimum and maximum viabilities, *k* is a parameter describing the steepness of the curve, and *χ*_0_ is a half-saturation constant. Thus, $(\alpha (\chi _{i}) \chi _{i})_{i = 1}^{S}$ represents living population sizes for this collection of taxa. Our underlying assumption is that the fraction of gene copies originating from living cells is a function only of the abundance of that gene. Because the functional form of this relationship is unknown for bacterial communities, we studied a model with many degrees of freedom (as parameterized by *ϕ*,*λ*,*χ*_0_, and *k*) to evaluate a wide range of community structures and dependencies between total DNA amounts and viabilities.

We performed 10^4^ iterations of this simulation procedure, independently drawing parameter values from uniform distributions (Additional file 2: Table S1); we then repeated this for each drawn parameter set, this time simulating the loss of a small number of abundant “light-sensitive” taxa by removing between 10 and 65 of the most abundant sequences from the SAD. This range was chosen because it reflected experimental outcomes (see “Results”). To simulate the sequencing of communities with these underlying SADs, we accounted for the fact that microbiome studies typically pool sequencing libraries in equal concentration of amplicons by performing size-biased random sampling of $(\alpha (\chi _{i}) \chi _{i})_{i = 1}^{S}$ at a fixed depth of 50,000 reads. This procedure generated abundance distributions meant to mimic those obtained from high-throughput sequencing, for pairs of viable communities that experienced the inactivation of dominant taxa but were otherwise identical. Model predictions were summarized using plots of the expected log10-fold apparent change in simulated sequence abundances for each community pair, as a function of the true abundances of those sequences.

## Results

### Light exposure alters total and living dust community structure

**Fig. 2:**
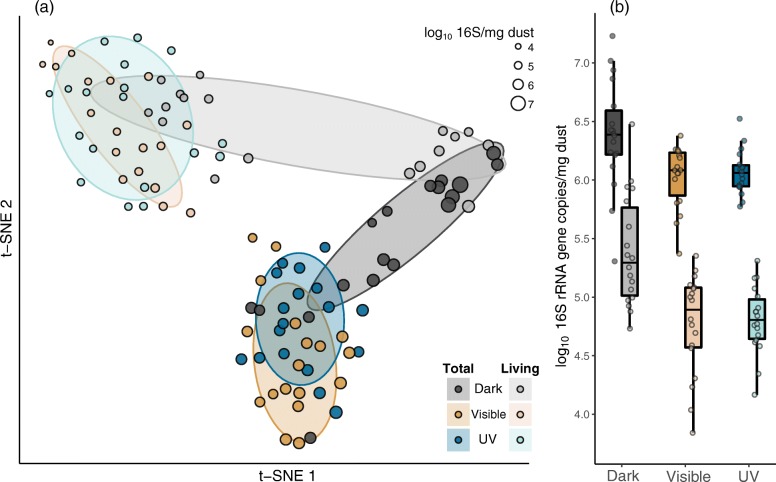
Effects of light on dust community *β*-diversity and microbial abundance. **a** t-distributed stochastic neighbor embedding (t-SNE; [52]) visualization of pairwise Canberra distances, calculated using log101+*x*-transformed RSV absolute abundances. Ellipses delineate treatment groups and represent one standard deviation from the group centroid. Points represent bacterial communities that are colored by their corresponding lighting regime: dark are gray, visible are gold, and ultraviolet are blue. The size of each point is scaled proportionately to the qPCR-based estimates of absolute bacterial abundances. Dark and light shades of each color indicate whether the sample represents the total or viable community respectively. **b** Boxplots of qPCR-based estimates of log10-transformed absolute abundance per milligram dust. The left and righthand boxes for each factor level correspond to the total and living bacterial abundances respectively. Colors are the same as in panel **a**

Absolute abundance-weighted *β*-diversities of total (i.e., the combined living and dead) communities varied significantly with treatment type (PERMANOVA; *R*^2^=0.116, *P*<0.001) indicating that patterns in bacterial abundances were in part determined by exposure to light and variation in particular wavelengths (Fig. 2a, dark-shaded points; Table 1). We did not detect an effect of mean daily light dosage (i.e., measurements from visible and UV light sensors) on community composition in either of the groups receiving light treatment. The largest differences in community composition were observed between dust communities experiencing darkness and those experiencing light per se—either visible (PERMANOVA; *R*^2^=0.111, adjusted *P*=0.002) or ultraviolet (*R*^2^=0.11, *P*=0.002) light wavelengths. We detected minor but significant differences between total communities experiencing visible and UV light (*R*^2^=0.032, *P*=0.043; compare *R*^2^ values), suggesting that different light wavelengths effected only minor changes in community RSV membership and abundance distributions for living and dead taxa.

**Table 1:** Results of pairwise PERMANOVA analyses of Canberra distance between treatment groups

Contrast	Total/living	*R* ^2^	adj. *P*
Dark-visible	Total	0.111	0.002
Dark-UV	Total	0.11	0.002
Visible-UV	Total	0.032	0.043
Dark-visible	Living	0.072	0.002
Dark-UV	Living	0.066	0.002
Visible-UV	Living	0.031	0.099

RSV features were weighted by their log101+*x*-transformed absolute abundances. The *Contrast* column indicates the pair of factor levels to which the statistics refer, and *Total/living* designates whether analysis was of the total (i.e., no PMA treatment) or living (i.e., PMA treated) components of the communities. Model results are provided in the *R*^2^ and Benjamini-Hochberg adjusted *P* values columns

The living (i.e., assayed using PMA) portion of each dust community exhibited similar quantitative *β*-diversity patterns (Fig. 2a, light-shaded points), with the exception of the contrast between the living visible and UV light communities (Table 1); we did not detect differences between these groups (*R*^2^=0.031, *P*=0.099), indicating that differences between bacterial dust communities experiencing visible and ultraviolet light wavelengths manifested primarily in abundances of dead members of those communities. Living dust communities were distinct from their combined living and dead counterparts on average, regardless of light treatment (PERMANOVA; *R*^2^=0.096, *P*<0.001). A multivariate dispersion analysis (permdisp2 procedure; Anderson, 2006) revealed that quantitative community compositions in the dark were more variable than in either visible or UV light microcosms (adjusted *P* values < 0.001; Fig. 2a, gray ellipses).

### Light exposure reduces living bacterial abundance

The qPCR-based estimates of total bacterial abundance (i.e., log10 16S rRNA gene sequence copy number for the combined living and dead bacteria) were marginally lower in visible (ANOVA; adjusted *P*=0.051) and ultraviolet (*P*=0.11; Fig. 2b) communities compared to dark ones. However, living bacterial abundance was significantly lower under both visible (Tukey’s post hoc test; *P*<0.001) and UV light (*P*<0.001; Fig. 2b). As a result, the estimated fraction of viable bacteria was highest in dark dust, on average. This fraction ranged from 0.4 to 73% across all communities, with an average of 12%, 6.8%, and 6.1% viability for dark, visible, and UV treatment groups respectively. Living bacterial abundances were comparable to previous estimates from built environment dust communities [64, 65]. Taken together, these results suggest that window-filtered light exposure, regardless of the particular transmittance profile, decreases the number of living bacteria in dust communities, but not necessarily total DNA amounts. We did not detect differences in living bacterial abundances between communities experiencing visible and ultraviolet light (Fig. 2b).

### Light exposure selects taxa derived from outdoor air

**Fig. 3:**
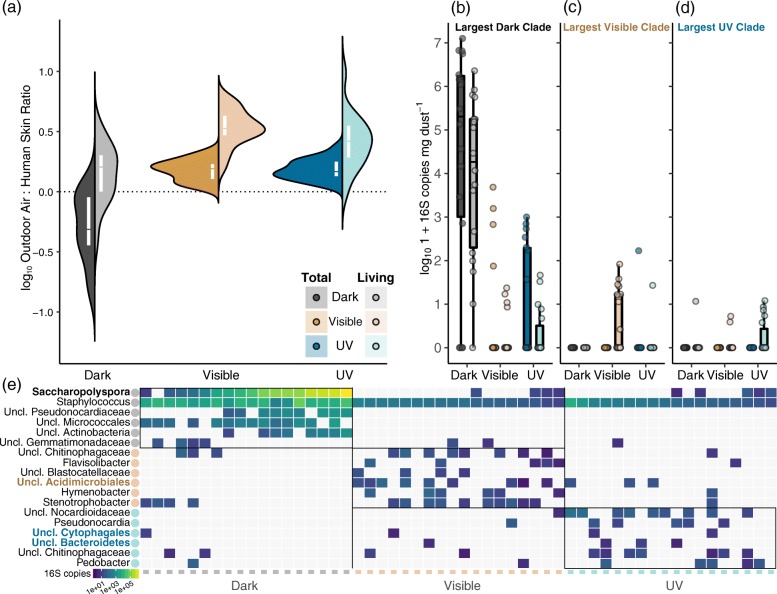
Responses of microbial taxa to light exposures. **a** Split violin plots summarizing results of microbial source tracking [56] analysis. The predicted sources of bacterial genera detected in experimental samples are summarized as log10*A*/*B* ratios, where *A* and *B* are the predicted proportions of genera originating from outdoor air and human skin respectively (69% of community genera on average). Values near 1 indicate that communities became more “outdoor air-like” (i.e., contain a plurality of outdoor air-sourced bacterial genera), while values near − 1 indicate that communities became more “human skin-like” by the experiment’s conclusion. White boxes denote group medians and first and third quartiles. **b**–**d** Boxplots of log101+*x*-transformed absolute abundances of the largest clades discriminating communities under each lighting treatment. These taxa are marked in bold in the rows of panel **e**. Colors and shades are the same as in Fig. 1. **e** Heatmap showing absolute abundances of viable discriminant taxa, detected using phylogenetic sLDA [59]. RSVs are aggregated based on the highest level of taxonomic classification. Warmer colors correspond to higher abundances; white tiles indicate those taxa were not detected in particular samples (columns). Columns are individual viable dust communities, where treatment group is indicated by the colored markers on the *x*-axis. Dark, visible, and ultraviolet-associated taxa are identified by colored circles near taxonomic labels and demarcated by black blocks. Matrix seriation was accomplished using principal components analysis

Bacterial source tracking [56] predicted that 69.2% of the genera that persisted in dust after the 90-day experiment originated from either human skin or outdoor air on average (Fig. 3a), a result that is consistent with prior predictions [66]. For dark, visible, and ultraviolet light groups respectively, the dust communities’ living fractions consisted of 15% ± 4.7%, 19.6% ± 1.3%, and 25% ± 2.2% skin-derived taxa and 24.2% ± 5.6%, 64.9% ± 2.1%, and 62.1% ± 2.1% (mean ± SEM) outdoor air-derived taxa on average. In contrast, fewer than 1% of genera on average were predicted to have originated from the human gut and soil habitats in our training set. Dust experiencing light comprised a significantly smaller proportion of predicted human skin-derived bacterial genera compared to dark communities (ANOVA; *P*<0.001) and instead contained a plurality of outdoor air-derived genera (Fig. 3a). A higher relative fraction of skin-derived bacterial genera was predicted for communities experiencing darkness, although these taxa consisted mainly of dead individuals (Fig. 3a, dark shades). The predicted proportion of outdoor air-sourced genera was higher in the living portion of all communities, and in particular those experiencing light (Fig. 3a, light shades).

### Related taxa are associated with darkness and light exposure

A phylogenetic tree-informed sparse discriminant analysis [59] identified a mixture of 12 small clades and 8 RSVs that strongly discriminated between dark, visible, and ultraviolet light dust communities (Fig. 3b–e; Additional file 3: Table S2) based on their feature loadings on the discriminating axis. The largest of these clades was a dark-associated group of 23 RSVs in the *Actinobacteria*. Of these, 18 RSVs were classified as members of the genus *Saccharopolyspora*. Members of this clade collectively accounted for an average of 30.1%, and as high as 90.1%, of dark communities and were highly abundant in the living portions of their respective communities (Fig. 3b, e). Together with this clade, a group of 12 RSVs classified as *Staphyloccocus* created a numeric gradient in community dominance in dark microcosms (Fig. 3e, top two rows). This gradient was responsible for the large amount of observed variability in dark communities (i.e., results of the permdisp2 analysis). These taxa were likewise rare in communities experiencing light, suggesting that these groups may be sensitive to light exposures conditional on their presence or initial abundance in dust inocula (see Additional file 1). The largest visible- and ultraviolet-associated clades each contained three RSVs in the *Acidimicrobiales* and *Cytophagales* respectively (Fig. 3c, d); these taxa were seldom detected in dark communities (Fig. 3e). These results indicate that our experimental light exposures led to the loss of a related set of numerically dominant, sensitive taxa and an apparent increase in the abundances of a small number of relatively rare, related RSVs (Fig. 3e; Additional file 3: Table S2).

### Sampling models identify potential mechanisms underlying empirical patterns

**Fig. 4:**
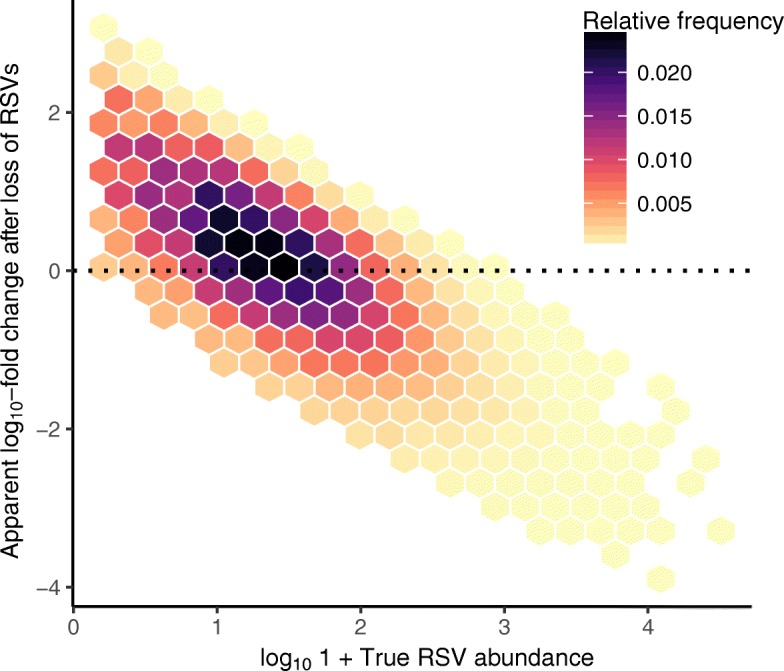
Simulations predict sampling artefacts following losses of abundant taxa. The relationship between a microorganism’s simulated absolute abundance in a community (*x*-axis), and its apparent log10-fold change in estimated abundance following the simulated loss of a small number of dominant taxa (*y*-axis). Predictions from 10^4^ iterations of the simulation procedure are summarized using 2D hexagonal bins; darker colors indicate higher frequency bins. Positive values on the *y*-axis indicate that abundances are underestimated in the presence of highly abundant RSVs, leading to an apparent positive increase in measured abundances following the loss of these RSVs. A common expected sampling artefact, whereby the loss of highly abundant RSVs, drives an apparent increase in the abundance of rare taxa is visible

Our sampling theory model generates two key results considering these empirical observations. First, the model predicts that an apparent increase in the abundances of a small number of very rare taxa can be expected to consistently occur under a wide range of potential conditions (Additional file 2: Table S1), if a few dominant taxa are inactivated and lost from the community (Fig. 4). Second, our model predicts that with the exception of these very rare RSVs, the majority of taxa that are sampled at a density below 500 gene copies per milligram of dust will not exhibit large apparent changes in estimated abundances (Fig. 4). In our experimental dataset, 99% of RSVs exhibited mean viable abundances below this threshold. Taken together with the fact that all dust inocula originated from a single homogenized pool (Additional file 1), results of our experiment and simulations point to two mechanisms that could have generated the observed increases in abundances of a few related bacterial taxa following lighting treatments (Fig. 3b–e). The first might be expected if these taxa increased in abundance and passed a threshold of detectability because of light exposures, for instance as a result of photosynthetic activity or the presence of other ecological or cellular mechanisms that facilitate population growth under lighting conditions. The second might be expected if these taxa exhibited *apparent* increases in abundance, due to the increased detection rate of very rare RSVs following the putative inactivation and loss of numerically dominant *Saccharopolyspora* and *Staphylococcus* by light (Fig. 3b, e)—a phenomenon that is predicted by the model (Fig. 4). Of course, these two possibilities are not mutually exclusive.

## Discussion

We observed marked differences in the compositions, abundances, and viabilities of microbial communities associated with household dust when exposure to daylight was experimentally disrupted (Figs. 2 and 3; Table 1). Communities associated with dust were more variable in darkness compared to those in the presence of daylight (Fig. 2a), which may indicate a convergence in community structures under regular disturbances [67, 68], in this case light disturbance [16]. Our results indicate that dust exposed to daylight contains smaller viable bacterial communities (Fig. 2b) that more strongly resemble outdoor air communities (Fig. 3a) and that the bactericidal effects of ordinary window-filtered sunlight may be similar to those achieved by ultraviolet light wavelengths for some taxa (Fig. 3b, e), but not for others (Fig. 3c, d).

Our experimental light exposures were associated with the loss of a related set of numerically dominant, potentially sensitive taxa (Fig. 3e, gray circles) and apparent increases in the abundances of a small number of rare taxa (Fig. 3e, gold and blue circles). However, we were unable to determine whether these apparent increases were due to metabolic activity and bacterial population growth under lighting conditions or the result of sampling artifacts arising from DNA sequencing. Photochemical transformation of organic materials due to exposures to visible or ultraviolet light wavelengths have been shown to increase bacterial growth rates in some ecosystems [69] and are at least one mechanism that could influence bacterial growth under strong daylighting. However, prior research indicates that many if not most built environment-associated bacteria require water activity greater than 95% for growth [64]—conditions that are significantly wetter than what was maintained in our microcosms. Instead, results of our experiment, sampling model, and prior studies point to the explanation that these apparent increases were artefacts resulting from the inactivation and loss of numerically dominant, light-sensitive taxa (Fig. 3e, gray circles). We hypothesize that when highly abundant community members like *Saccharopolyspora* and *Staphyloccocus* were lost, the underlying taxonomic abundance distribution was truncated in a way that mitigated our inability to detect very rare RSVs. Sampling theory provides a path to further understand what drives the underlying structure of microbiomes by establishing null expectations for ecological patterns [3, 62, 70]; microbiome studies will benefit from a continued consideration of quantitative theories that explicitly account for the technological limitations and biases surrounding the detection of rare microorganisms from environmental DNA [71].

The most diverse and abundant group of organisms associated with dark dust contained members of the genus *Saccharopolyspora*, which have been previously associated with soils and buildings in rural areas [72], and built environment-mediated respiratory diseases [73, 74]. The observation that these dominant RSVs were largely absent or rare in daylit dust provides some evidence to the hypothesis [21] that sunlight may be used to selectively limit the viabilities of microorganisms in buildings like hospitals, although we are not able to determine the pathogenic potential of any of the bacteria detected in this study. Additional experiments are needed, to determine the microbicidal potential of light exposures under a wider range of conditions, especially in conjunction with the enhanced indoor microbial growth rates reported under elevated water availability [64, 75] and with an explicit focus on known pathogenic microorganisms including viruses, fungi, archaea, and protists. Interactions between sunlight and population sizes have been observed for a small number of viral, [76] fungal [77], and protozoan [78] taxa in other systems, but these relationships have not yet been uncovered for holistic dust communities that comprise multiple microbial kingdoms in real buildings [7]. Experimental studies that include detailed time series measurements are also needed to characterize the transient dynamics and mechanisms underlying sunlight-induced changes in dust microbial communities, which may exhibit phylogenetic signals or depend on functional genes related to photosynthesis, photoreactivation and repair [79], and oxidative stress [80].

We used a model system to study the effects of light exposure on the structure of microbial dust communities, although we expect many of the results observed in this study to apply to real built environments. Our microcosms were designed to approximate conditions in real buildings, including temperatures, reflectances, humidities, and transmittances. While the microcosms used here permit more control compared to typical built environment microbiome studies, these systems are still idealized representations of human-occupied spaces. Our experiment was limited in that it characterized features of the dust microbiome across a relatively narrow range of light dosages. We aimed for dosages relevant to well-daylit buildings, but there are many architectural and geographical instances that produce lower or higher dosages than examined here that may merit additional study. Our microcosms were south-facing and therefore experienced the greatest possible daily exposures. Other latitudes, altitudes, climate zones, building orientations, and obstructions (e.g., trees) would indeed change exposures raising the possibility of linkages between the spatial context of buildings, design decisions that impact the transmittance of light, geographical or seasonal variation in sunlight availability, and the structure of indoor dust microbial communities.

## Conclusions

Our experiment suggests that the use of ultraviolet-filtering glazing, that is found in many if not most buildings, may not be a significant shaper of indoor dust communities as originally anticipated, in comparison to glazing that transmits ultraviolet wavelengths. It also suggests that architects and lighting professionals designing building facades and rooms with more or less access to daylight may play a role in influencing the microbial communities of indoor dust. However, the impacts of daylight exposure on the dust microbiome uncovered here, relative to other factors like building occupancy, geography, ventilation, and humidity [3, 5, 6, 27, 64, 75], remain unclear, emphasizing the pressing need for controlled indoor experiments that are designed to disentangle the likely complex and context-dependent relationships among covarying abiotic drivers and the dust microbiome.

## Additional files

Additional file 1 **Supplementary Information.** Additional details on training data used for microbial source tracking and dust homogenization techniques. (PDF 68 kb)

Additional file 2 **Table S1.** Table describing the range of parameter values considered for sampling model simulations. (XLSX 29 kb)

Additional file 3 **Table S2.** Table describing the taxonomy of RSVs identified by sLDA analysis. (XLSX 10 kb)

## Abbreviations

PCR: Polymerase chain reaction
