# Supplementary material for: Daylight exposure modulates bacterial communities associated with household dust
Source: Microbiome. 2018 Oct 18;6:175. doi: 10.1186/s40168-018-0559-4 (PMC6193304; doi:10.1186/s40168-018-0559-4)
Supplement: Supplementary file 1 — Supplementary Information. Additional details on training data used for microbial source tracking and dust homogenization techniques. (PDF 68 kb) [file 40168_2018_559_MOESM1_ESM.pdf]

Supplementary information: Daylight exposure modulates bacterial communities associated with household dust

Ashkaan K Fahimipour[1,*], Erica M Hartmann[1,2], Andrew Siemens[1], Jeff Kline[1,3], David A Levin[4], Hannah Wilson[1], Clarisse M. Betancourt-Román[1], G.Z. Brown[1,3], Mark Fretz[3], Dale Northcutt[1,3], Kyla N Siemens[1], Curtis Huttenhower[5], Jessica L Green[1,6], and Kevin Van Den Wymelenberg[1,3]

[1]Biology & the Built Environment Center, University of Oregon, Eugene, Oregon, USA

[2]Dept. of Civil and Environmental Engineering, Northwestern University, Chicago, Illinois, USA

[3]Energy Studies in Buildings Laboratory, University of Oregon, Eugene, Oregon, USA

[4]Dept. of Mathematics, University of Oregon, Eugene, Oregon, USA

[5]Dept. of Biostatistics, Harvard T.H. Chan School of Public Health, Boston, MA, USA

[6]Santa Fe Institute, Santa Fe, NM, USA

Additional details on data used for source tracking

Outdoor air settling dishes

On 2016 Aug 2, three 150 $\times$ 15 mm sterile petri dishes were placed outside of 27 homes in Eugene, OR, USA to accumulate settled outdoor air particles. After a one week period, petri dishes were collected, sealed with film, placed in sterile Nasco (Fort Atkinson, WI, USA) Whirl-Paks, and stored at -20°C until collection at all sites was completed. For each set of three petri dishes, 100 μL of 1x PBS (Phosphate Buffered Saline, pH 7.2) was added to each petri dish, and then all three were thoroughly and systematically swabbed with a single sterile FLOQSwab (Specimen Collection Swab, 80mm, Copan Diagnostics). The swab was then placed in a sterile bead tube (MoBio 0.1 mm glass bead 2 mL tube) and stored at -80°C.

The DNA from dish samples was manually extracted using the MoBio PowerLyzer PowerSoil DNA Isolation Kit (MoBio, Carlsbad, CA, USA) according to manufacturer's instructions. DNA were amplified in a PCR enrichment

of the V3-V4 (319F-806R) regions of the 16S rRNA gene using dual-barcoded primers, designed by the University of Oregon Genomics Core Facility to be compatible with current Illumina sequencing platforms. PCRs were purified with a modified bead-based DNA clean-up protocol using Mag-Bind RxnPure Plus by Omega Bio-tek, quantified using Quant-iT dsDNA assay kit, and pooled with equal concentrations of amplicons using an Eppendorf epMotion 5075 robot. Libraries were sequenced on an Illumina MiSeq generating 250 bp paired end reads, which were processed using the DADA2 statistical algorithm with the Silva version 128 reference database, as described in the main text.

Human skin swabs

This study and its associated research protocols were approved by the IRB at the University of Oregon on December 23rd, 2013. Healthy, adults aged 18-35 were recruited from the Eugene, OR, USA area. The eligibility requirements for participation included that the individual was free of any skin conditions or infections and had not received any antibiotics within the last 6 months. Subjects were instructed to refrain from bathing and applying topicals to the skin for 12 hours prior to the censuses. To sample skin communities, swabs were dipped into sterile saline solution (0.15 M NaCl; 0.1% Tween20) and rotated on forearm and calf skin sites for *ca.* 15 seconds.

The DNA from swabs was manually extracted using the MoBio PowerLyzer PowerSoil DNA Isolation Kit (MoBio, Carlsbad, CA, USA) according to manufacturer's instructions. DNA were amplified in a PCR enrichment of the V4 (515F-806R) regions of the 16S rRNA described by (Walters et al., 2016), following the 16S rRNA amplification protocols by the Earth Microbiome Project (earthmicrobiome.org). PCRs were purified with a modified bead-based DNA clean-up protocol using Mag-Bind RxnPure Plus by Omega Bio-tek, quantified using Quant-iT dsDNA assay kit, and pooled with equal concentrations of amplicons using an Eppendorf epMotion 5075 robot. Libraries were sequenced on an Illumina MiSeq generating 150 bp paired end reads. All data were processed using the DADA2 statistical algorithm with the Silva version 128 reference database, as described in the main text.

Analysis of dust homogenization procedure

Following the addition of initial experimental inocula to microcosms, surplus dust from the homogenized pool (see *Methods*) was stored under ambient indoor conditions in a sealed container that prevented light exposures ("archived dust"). Although the effects of this storage method on the associated microbiome are unknown, archived dust was used to quantify the variation in bacterial community compositions that could be expected from repeated samples

taken from the same homogenized dust pool. On 2016 March 18, five aliquots weighing 0.25g were taken from the archived dust pool and subdivided into two equal parts. One part was treated with propidium monoazide (PMA), and then both were processed, sequenced, and quantified using real-time qPCR as in the main text. Raw sequencing data were processed using the same DADA2 workflow described in the *Methods* (Callahan et al., 2016) and likewise scaled according to qPCR-based estimates of 16S rRNA gene copy numbers, to estimate absolute abundances of ribosomal sequence variants (RSVs) in these subsamples.

A multivariate dispersion analysis with ANOVA and Tukey's post hoc test (permdisp2 procedure; Anderson, 2006) was used to compare within-group β-diversities (pairwise Canberra distances of $\log_{10} 1 + x$-transformed abundances) among bacterial communities from experimental microcosms to those obtained by subsampling directly from a homogenized dust pool. When the total and living portions communities in each treatment group were compared (Fig. S1), we consistently detected less variation in community compositions (i.e., β-diversities) in the archived dust samples than in the experimental microcosm communities (all P-values < 0.009). Two key conclusions can be taken from this result in light of experimental outcomes. First, the lower variation in both total and living archived dust communities suggests that the laboratory homogenization procedure generates repeatable dust inocula with low variation in initial conditions relative to the final communities. Second, this result indicates that, despite the relative consistency of these samples, small variation in the abundances of RSVs are impossible to avoid and likely characterized initial experimental inocula. Thus, small variation in the initial experimental communities ought to be expected, and it is conceivable that this variation could have influenced the outcomes of experimental light exposures. In particular, we suspect that the numerical gradient between *Saccharopolyspora* and *Staphyloccocus* reported in *Results* (Fig. 3e) was at least in part due to small variation in the initial abundances of these taxa. Detailed time series measurements are a pressing need for future built environment experiments, as these data will be necessary for characterizing the transient dynamics of microbial inactivation by daylight on indoor dust.

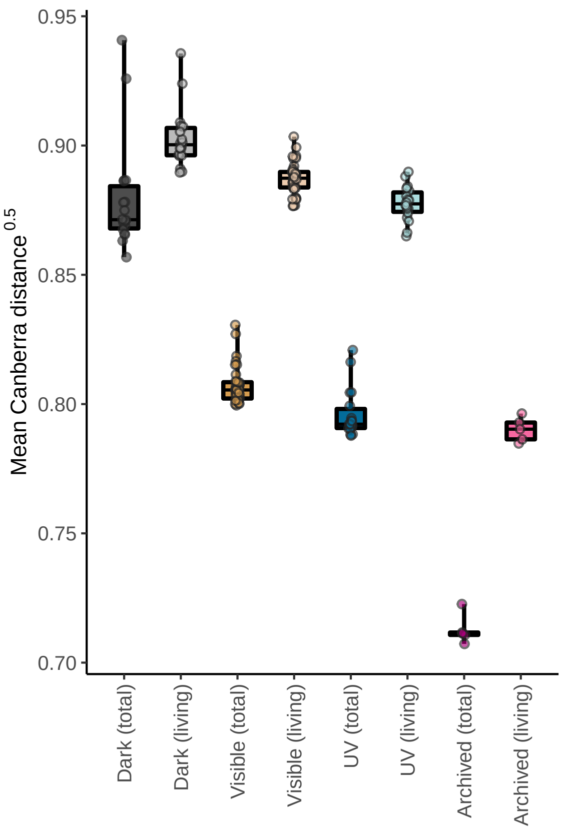

Figure 1: Within-group variation in community composition for the total (darker shades) and living (lighter shades) portions of the experimental and archived dust communities. For all contrasts among living and total communities, variation in archived dust bacterial community compositions (pairwise Canberra distances) were lower than experimental communities (permdisp2 procedure; all P-values < 0.009).

References

Anderson, M. J. (2006). Distance-based tests for homogeneity of multivariate dispersions. *Biometrics*, 62(1):245–253.

Callahan, B. J., McMurdie, P. J., Rosen, M. J., Han, A. W., Johnson, A. J. A., and Holmes, S. P. (2016). Dada2: high-resolution sample inference from illumina amplicon data. *Nature methods*.

Walters, W., Hyde, E. R., Berg-Lyons, D., Ackermann, G., Humphrey, G., Parada, A., Gilbert, J. A., Jansson, J. K., Caporaso, J. G., Fuhrman, J. A., et al. (2016). Improved bacterial 16s rrna gene (v4 and v4-5) and fungal internal transcribed spacer marker gene primers for microbial community surveys. *mSystems*, 1(1):e00009–15.
